# Evidence on the ecological and physical effects of built structures in shallow, tropical coral reefs: a systematic map

**DOI:** 10.1186/s13750-024-00336-3

**Published:** 2024-05-14

**Authors:** Avery B. Paxton, Iris R. Foxfoot, Christina Cutshaw, D’amy N. Steward, Leanne Poussard, Trevor N. Riley, Todd M. Swannack, Candice D. Piercy, Safra Altman, Brandon J. Puckett, Curt D. Storlazzi, T. Shay Viehman

**Affiliations:** 1 National Centers for Coastal Ocean Science, National Ocean Service, National Oceanic and Atmospheric Administration, 101 Pivers Island Road, Beaufort, NC 28516 USA; 2 U.S. Army Engineer Research and Development Center, 3909 Halls Ferry Road, Vicksburg, MS 39180 USA; 3 UIC Government Services, 6564 Loisdale Ct #900, Springfield, VA 22150 USA; 4 CSS-Inc, 10301 Democracy Lane, Suite 300, Fairfax, VA 22030 USA; 5 Central Library, Office of Science Support, Oceanic and Atmospheric Research, National Oceanic and Atmospheric Administration, 1315 East‑West Highway, Silver Spring, MD 20910 USA; 6 Pacific Coastal and Marine Science Center, U.S. Geological Survey, 2885 Mission Street, Santa Cruz, CA 95060 USA

**Keywords:** Artificial structures, Designed habitat, Coastal protection, Coastal restoration, Coastal mitigation, Eco-engineering, Natural infrastructure, Nature-based solutions, Nature-inspired designs

## Abstract

**Background:**

Shallow, tropical coral reefs face compounding threats from climate change, habitat degradation due to coastal development and pollution, impacts from storms and sea-level rise, and pulse disturbances like blast fishing, mining, dredging, and ship groundings that reduce reef height and complexity. One approach toward restoring coral reef physical structure from such impacts is deploying built structures of artificial, natural, or hybrid (both artificial and natural) origin. Built structures range from designed modules and repurposed materials to underwater sculptures and intentionally placed natural rocks. Restoration practitioners and coastal managers increasingly consider incorporating – and in many cases have already begun to incorporate – built structures into coral reef-related applications, yet synthesized evidence on the ecological (coral-related; e.g., coral growth, coral survival) and physical performance of built structures in coral ecosystems across a variety of contexts (e.g., restoration, coastal protection, mitigation, tourism) is not readily available to guide decisions. To help fill this gap and inform management decisions, we systematically mapped the global distribution and abundance of published evidence on the ecological (coral-related) and physical performance of built structure interventions in shallow (≤ 30 m), tropical (35°N to 35°S) coral ecosystems.

**Methods:**

To identify potentially relevant articles, we used predefined and tested strategies to search two indexing platforms, one bibliographic database, two open discovery citation indexes, one web-based search engine, one novel literature discovery tool, 19 organizational websites, and information requested from stakeholders. Discovered articles were screened according to preset eligibility criteria first by title and abstract and second by full text. Articles included during full text screening were coded to extract metadata following a predefined framework. We analyzed and visualized the evidence base to answer our primary and secondary research questions and to identify knowledge clusters and gaps. Findings are reported in a narrative synthesis.

**Results:**

Our search discovered > 20,000 potentially relevant unique articles, of which 258 were included in the systematic map. The evidence base spans 50 countries, and the volume of evidence increased over the past five decades. Built structures were most commonly installed for coral restoration (61%) or coastal protection (12%). Structures were predominately characterized as artificial (87%), with fewer hybrid or natural interventions. Evidence clusters existed for intentionally designed artificial structures and outcomes associated with coral-related ecological performance, including coral mortality, growth, recruitment, cover, and diversity. Pronounced evidence gaps occurred at the intersection of several ecological coral-related performance outcomes (e.g., connectivity, microbiome) across all types of built structures; gaps also existed across most ecological coral-related outcomes for artwork and repurposed artificial structures. Physical performance of built structures was most frequently evaluated for outcomes related to waves (*n* = 14) and sediment and morphology (*n* = 11) with pervasive evidence gaps across other outcomes like storm surge and water level.

**Conclusions:**

While the systematic map highlighted several evidence clusters, it also revealed pronounced evidence gaps surrounding the coral-related ecological and physical performance of built structures in coral ecosystems. The compiled evidence base will help inform policy, management, and future consideration of built structures in reef-related applications, including habitat restoration, environmental mitigation, and coastal protection. Map findings also point to promising future research avenues, such as investigating seascape-scale ecological effects of and the physical performance of built structures.

**Supplementary Information:**

The online version contains supplementary material available at 10.1186/s13750-024-00336-3.

## Background

Coral reefs provide extensive ecosystem services, including biodiversity benefits, coastal protection, and fisheries provisioning [1], yet face global declines from multiple threats [2, 3]. Local threats include those from habitat degradation often linked to coastal development [4], overfishing [5], and pollution [6–8], as well as from disturbances like blast fishing [9], coral mining [10], dredging [11], and ship groundings [12]. Global impacts from climate change include coral mortality from ocean warming and associated bleaching [13], disease [14], and ocean acidification [15]. Climate change is also increasing the severity and frequency of storms that can further degrade coral reefs by breaking and dislodging coral [16] and increasing sedimentation, which reduces the potential for successful coral recruitment [17, 18].

Strategies to slow or reverse declines in coral reefs often include restoration, such as direct transplantation of corals or larval enhancement [19]. Coastal managers and restoration practitioners are increasingly considering the incorporation of built structures into coral restoration design and implementation [20]. Here, we define built structures as those that have been engineered, designed, created, built, or constructed using artificial, hybrid, or natural materials. We define restoration broadly, following the UN Decade on Ecosystem Restoration, as “efforts to prevent, halt, or reverse the degradation of ecosystems” [21, 22]. For coral reefs, this definition includes partial and holistic ecosystem recovery and thus actions aimed towards returning reefs to a historical state or creating new reefs [20, 23]. Built structures have a centuries-long history of being deployed in the seascape for multiple objectives. For example, artificial reefs have been purposely sunk since the 1600s [24] to increase fishing yield, provide recreation opportunities, and conduct scientific research experiments, but in select cases have also been used specifically to restore coral reefs by creating, replacing, supplementing, enhancing, or stabilizing structured habitat [25, 26]. These intentionally deployed structures include those that have been repurposed from their original uses (e.g., concrete pipes originally used in construction), as well as modules designed for particular contexts, such as restoration of target coral species or specific seascape settings [27, 28]. In the past decade, underwater artwork installations have grown in popularity, as artwork and sculpture gardens have been commissioned and implemented to help restore corals and generate locations for recreational divers to enjoy [29, 30] (1,000 Mermaids Artificial Reef Project, https://1000mermaids.com/).

Built structures have also been used for environmental mitigation and coastal protection purposes. Structures installed for environmental mitigation seek to address impacts from disturbances like blast fishing, ship grounding, coral mining, dredging, and storms, which can reduce reef height and complexity and create excess amounts of rubble that prevent survival of coral recruits [31, 32]. In these instances, natural rock, hybrid structures (e.g., rock with cement, rock with mesh net), or human-made structures (e.g., concrete) have been deployed to provide habitat [33], stabilize rubble, and allow for recruit survival [31]. The role of coral reefs in providing coastal protection benefits has become increasingly apparent, as coral reefs can reduce wave energy by up to ∼97% where present [34] and thus provide ∼$1.8 billion in hazard risk reduction benefits per year in the U.S. alone [35, 36]. New initiatives have been launched to design engineered reefs for coastal protection. In Grenada, modular engineered structures were deployed to help reduce coastal erosion and flooding [37], whereas in southeast India, trapezoidal artificial modules were deployed to dissipate wave energy [38]. Newly funded Department of Defense projects in the U.S. aim to create hybrid reef structures that incorporate artificial (e.g., “gray) elements and natural (e.g., “green”) elements to mitigate flooding, erosion, and storm damage (Reefense, https://www.darpa.mil/program/reefense).

Despite the history and increasing consideration of built structures for coral restoration and related applications like environmental mitigation and coastal protection, questions remain regarding how built structures should be considered in management and restoration decisions. For instance, how do built structures relate to coral growth, cover, and condition, and how do built structures relate to wave energy and storm surge? Central to these questions is that the global evidence base regarding the use and performance of built structures has not been collated or synthesized; but see syntheses for particular contexts, such as artificial reefs [26], substrate stabilization [31], and 3D technology for reef structures [39]. The lack of broadly synthesized evidence presents barriers to implementing management and policy decisions regarding future use of built structures in coral reef systems. Without synthesized evidence, it is more challenging for decision makers to rigorously and reproducibly evaluate the appropriateness of built structures for providing restorative, mitigative, or protective ecological outcomes in particular seascape settings.

The goal of this study was to collate evidence on coral-related ecological performance, as well as the physical performance, of built structure interventions in shallow, tropical coral reef settings. We use “built structures” as an umbrella term encompassing structures of artificial, hybrid, or natural origin. We use “hybrid” to describe structures that have both artificial (i.e., “gray”) and natural (i.e., “green) elements. This synthesis of knowledge will help inform practice for built structure design, siting, and implementation, including for nature-based solutions that can help address societal and ecological challenges, such as those related to scaling up and achieving successful habitat restoration and environmental mitigation, in coral reef settings. Because built structures have been used for multiple applications related to tropical coral reefs, such as for restoration, coastal protection, and environmental mitigation, we included evidence from these diverse bodies of literature. This will ensure that our synthesis stems from the most comprehensive body of relevant literature and will help ensure that findings from our synthesis can be used to help guide management decisions regarding the design, siting, and implementation of built structures in coral reef settings.

### Stakeholder engagement

This systematic map was a joint effort by scientists from the National Oceanic and Atmospheric Administration (NOAA) National Centers for Coastal Ocean Science (NCCOS), the U.S. Army Corps of Engineers (USACE) Engineer Research and Development Center (ERDC), and the U.S. Geological Survey (USGS) Coastal and Marine Hazards and Resources Program (CMHRP). The core team of scientists from NOAA, USACE, and USGS developed the systematic map protocol to address stakeholder needs [40]. During the mapping process, we consulted additional stakeholders and scientists from the U.S. and internationally to ensure that international sources of primary literature were incorporated into the map.

## Objective of the review

The objective of this systematic map was to document the global evidence base on the ecological and physical performance of built structures in shallow, tropical coral reef settings. The systematic map also aimed to summarize how evidence differs by built structure qualities, such as the type and material of intervention, as well as the goal and seascape setting.

The primary research question for the systematic map was: What is the distribution and abundance of evidence on the ecological and physical performance of built structures in shallow, tropical coral reef systems? The components of this primary question are:

- **Population**: Coral reefs located in shallow, tropical coastal environments (≤ 30 m depth, 35^o^N to 35^o^S latitude).
- **Intervention**: Built structures of artificial, hybrid, or natural origin established in coral systems.
- **Comparator**: Studies that include a spatial or temporal comparator (presence vs. absence of built structure intervention, before vs. after built structure intervention, different types of built structure interventions, etc.) were included. Articles without comparators, but that did have qualifying outcomes measured at one point in time or at one spatial location, could be included because they provided valuable “snapshot” evidence. See ‘eligibility criteria’ for additional information.
- **Outcome**: Ecological (coral-related – e.g., coral recruitment, coral mortality) or physical (e.g., waves, current, flooding) performance outcomes associated with built structure intervention.
- **Study type**: Experimental, observational, or modeling studies with quantitative data on ecological or physical outcomes associated with the intervention. Studies could be conducted in the field or lab settings.

The evidence base used to answer the primary question also allowed us to investigate the following secondary questions:

- How does the distribution and abundance of evidence on the performance of built structures used in coral reef-related applications differ by intervention type (e.g., artificial – designed structures, artificial – repurposed structures, artificial – artwork, hybrid structures of artificial and natural origin, and natural structures of geologic origin)?
- For which materials (e.g., concrete, metal, rock, fiberglass) and types (e.g., reef modules, concrete pipes, natural rock, mesh over rubble) of built structures has the performance been evaluated?
- For which ecological and physical outcomes has the performance of built structures used in coral reef-related applications been evaluated?
- How does the distribution and abundance of evidence on built structures differ by intervention goal or context (e.g., restoration, environmental mitigation, coastal protection, tourism), seascape setting (e.g., depth, energetic environment, relative location on reef), spatial scale, and geographic region?

## Methods

The protocol for this systematic map was published in *Environmental Evidence* in 2023 [40]. There were two deviations from the protocol. First, we conducted the database search following the initial round of peer-review but prior to protocol final acceptance and publication. Specifically, we received the initial peer-reviews of the systematic map protocol on April 7, 2023. Because the peer-reviews indicated that no changes to our search string would be required for mapping, we conducted the database search shortly thereafter from April 30, 2023 to May 3, 2023. We received a second round of protocol peer-reviews on July 25, 2023, which also required no changes to the database search. The protocol was officially accepted on August 10, 2023. We made the decision to execute the database search prior to final acceptance and publication of the protocol manuscript due to project timeline and staffing constraints but were prepared to modify the search strategy and rerun the search should any concerns or issues have been found during the peer-review process. We understood that this was a risk; however, we were confident with the quality of the search and that this had been confirmed in the initial round of the protocol peer-review process. Second, the organizational website Reef Base was not searched because the website was inaccessible. The systematic map followed evidence synthesis standards from the Collaboration for Environmental Evidence [41] and used the RepOrting standards for Systematic Evidence Synthesis (ROSES) [42] (Additional File 1).

### Search for articles

The search for articles was conducted from April 30, 2023 to May 3, 2023 in Web of Science, Scopus, Lens, Dimensions, ProQuest, Google Scholar, and Inciteful. Organizational website searches were conducted during September 2023. All searches were performed in English. The geographic scope was global. There were no temporal scope constraints.

#### Search string

The search string was created using Web of Science syntax. The search string syntax was adapted for the other sources (Additional File 2). See the protocol [40] for details of search string development and testing.

Population terms: coral*

*AND*

Intervention terms: artificial* OR gray* OR grey* OR engineer* OR hybrid* OR design* OR construct* OR install* OR built* OR build* OR deploy* OR sink* OR sunk* OR sank* OR modul* OR structur* OR biorock* OR concrete* OR “reef ball*” OR ecoreef* OR “eco reef*” OR “eco-reef*” OR “mars assisted reef restoration*” OR “mineral accretion*” OR tetrapod* OR tetrahedron* OR trapezoid* OR “reef mattress*” OR “reef unit*” OR “reef star*” OR “reef spider*” OR print* OR fabricat* OR rebar* OR artwork* OR sculpt* OR monument* OR decommission* OR ship* OR pipe* OR tire* OR tyre* OR bridge* OR repurpose* OR “re-purpose*” OR eternal* OR “self-healing*” OR “self healing*” OR terracotta* OR clay* OR ceramic* OR tile* OR “human-made*” OR “human made*” OR “man-made*” OR “man made*” OR manmade* OR biomimic* OR mimic* OR “biogenic structure*” OR “biogenic material*” OR limestone* OR boulder* OR rubble* OR cobble* OR rock* OR unconsolidate* OR “natural material*” OR “natural structure*” OR “natural reef*” OR “nature based solution*” OR “nature based strateg*” OR “nature based defen$e*” OR “nature based protection*” OR “nature based coastal” OR “nature based shoreline*” OR “nature based mitigation” OR infrastructure* OR “nature based infrastructure” OR “hybrid infrastructure” OR “hybrid technique*” OR “natural climate solution*” OR “natural infrastructure” OR “eco* engineer*” OR “eco-engineer*” OR ecoengineer* OR “eco* friendly engineering” OR “ecosystem friendly engineering” OR bioengineer* OR “blue engineering” OR “green engineering” OR “building with nature” OR “engineering with nature” OR “working with nature” OR “nature derived solution*” OR “nature based feature*” OR “nature inspired solution*” OR “nature inclusive design*” OR “nature inspired design*” OR “nature derived design*” OR “ecosystem* based adaptation*” OR “ecosystem* based mitigation” OR “disaster risk reduction” OR “coastal defen$e*” OR “blue infrastructure” OR “green infrastructure” OR “ecosystem based disaster risk reduction” OR “hazard* mitigation*” OR “hazard* risk*” OR “coast* protect*” OR “reefense” OR “x-reef*” OR stabili$*

*AND*

restor* OR mitig* OR enhanc* OR creat* OR supplement* OR rehabilitat* OR protect* OR “damage reduc*” OR “risk reduc*” OR attenuat* OR “coastal defen$e*” OR stabili$* OR recover* OR resilienc* OR “hazard risk*” OR conserv* OR infrastructure* OR “nature based*” OR engineer* OR recreat* OR touris* OR dredg* OR ground* OR “blast fish*” OR mining*

*AND*

Ecological outcome terms: grow* OR cover* OR communit* OR rich* OR divers* OR surviv* OR settle* OR dens* OR recruit* OR abund* OR size* OR coloniz* OR rugos* OR complexit* OR “surface area*” OR volume* OR connectiv* OR dispers* OR disease* OR mortalit* OR fragment* OR breakage* OR condition* OR bleach* OR succession* OR bioaccumul* OR “bio-accumul*” OR “chemical concentrat*” OR “biological interact*” OR succession* OR competit* OR predat* OR mutual* OR commensal* OR facilitat* OR parasit* OR omniv* OR zooplank* OR herbiv* OR piscivor* OR invasiv* OR invad* OR calcific* OR skelet* OR accret* OR gene OR genes OR genetic* OR corridor* OR distribut* OR composit* OR tissue* OR extens* OR zooxanth* OR symbio* OR microb* OR microorgan* OR “micro organ*” OR physiol* OR respir* OR photosynth* OR photopigm* OR histol* OR metabol* OR friction* OR bathy* OR curv* OR aspect* OR slop* OR fertiliz* OR embryo* OR planulat* OR health* OR diamet* OR “coral watch” OR stabili$* OR struct*

*OR*

Physical outcome terms: wave* OR current* OR friction* OR rough* OR flood* OR inundat* OR protect* OR forc* OR eros* OR erod* OR “storm surge*” OR break* OR sediment* OR attenuat* OR energ* OR flux* OR reduc* OR mitig* OR defen* OR tide* OR tidal* OR “sea level*” OR “water level*” OR elevat* OR shoreline* OR scour* OR damp* OR amplif* OR expos* OR circulat* OR fetch* OR buffer* OR stress* OR velocit* OR speed* OR direction* OR magnitud* OR redistribut* OR compact* OR consolid* OR trap* OR retain* OR retent*

#### Comprehensiveness of the search

We identified 21 benchmarking articles to test against the search string in Web of Science and estimate the comprehensiveness of our search string. These articles were sourced from subject matter experts and our core research team. Of the 21 articles, 18 were indexed and thus available in Web of Science (Additional File 3). The three articles that were not indexed in Web of Science were found in other databases [43–45]. Our search string found 16 of the 18 articles that were indexed in Web of Science. The two articles that were indexed in Web of Science but were unable to be identified with our search string did not include terms related to the intervention in the title or abstract and so were thus undetectable. These two articles had been provided by the synthesis team and had case studies embedded within them. See the protocol for additional details on benchmarking [40].

#### Indexing platforms

We searched two indexing platforms, Web of Science (WOS) Core Collection and Scopus. The WOS search was conducted with five indexes:

- SCI-Expanded (1980 - present).
- SSCI (1980 - present).
- CPCI-S (1990 - present).
- CPCI-SSH (1990 - present).
- ESCI (2018 - present).

Document types searched included articles, proceedings papers, early access, and data papers. The Duke University subscription was used for the WOS search. The Scopus search was conducted using the Duke University subscription with no filters.

#### Bibliographic databases

We searched the bibliographic database ProQuest Earth, Atmospheric and Aquatic Sciences Collection. Search indexes were.

- Aquatic Sciences and Fisheries Abstracts.
- Meteorological and Geoastrophysical Abstracts.
- Earth, Atmospheric, and Aquatic Sciences Database.
- Oceanic Abstracts.

Source types included scholarly journals, dissertations and theses, conference papers and proceedings, and reports. The Duke University subscription was used.

#### Open discovery citation indexes

Two open discovery citation indexes, LENS and Dimensions, were searched. The LENS search (lens.org) included four indexes:

- CORE.
- Crossref.
- PubMed.
- Microsoft Academic.

LENS was searched for journal articles, conference proceeding articles, conference proceedings, dissertations, and reports. Dimensions was searched for articles and proceedings. No subscription was required for either.

#### Web-based search engine

We searched Google Scholar using Publish or Perish version 8 [46]. The simplified search string used for Google Scholar, due to limitations of this search engine, was:

(coral AND reef) AND (artificial OR gray OR grey OR engineer OR hybrid OR design OR construct OR install OR built OR build OR deploy OR infrastructure OR “nature based solution” OR “nature inspired design”).

We conducted a title search for up to 1,000 articles, as per guidance from Haddaway, Collins, Coughlin and Kirk [47].

#### Novel literature discovery tool

We searched Inciteful, an online novel literature discovery tool [48], to find additional articles. Inciteful was seeded using a .RIS file of the benchmarking articles, and the tool returned up to 1,000 most similar papers.

#### Organizational websites

Searches were conducted in the following 19 organizational websites from September 12–21, 2023:

- Conservation International: https://www.conservation.org/.
- Coral Reef Alliance: https://coral.org/en/.
- Florida Department of Environmental Protection: https://floridadep.gov/.
- Global Coral Reef Alliance: https://www.globalcoral.org/.
- International Union for Conservation of Nature: https://www.iucn.org/.
- National Oceanic and Atmospheric Administration: https://www.noaa.gov/.
- Sea Grant: https://seagrant.noaa.gov/.
- The Nature Conservancy: https://www.nature.org/.
- United Nations Decade on Restoration: https://www.decadeonrestoration.org/.
- United Nations Development Programme: https://www.undp.org/.
- United Nations Environment Programme: https://www.unep.org/.
- United Nations Environment Programme World Conservation Monitoring Center: https://resources.unep-wcmc.org/.
- U.S. Army Corps of Engineers: https://www.usace.army.mil/.
- U.S. Geological Survey: https://www.usgs.gov/.
- U.S. Fish and Wildlife Service: https://www.fws.gov/.
- Wildlife Conservation Society: https://library.wcs.org/.
- World Bank: https://www.worldbank.org.
- World Resources Institute: https://www.wri.org/.
- World Wildlife Fund: https://www.worldwildlife.org/.

These searches were conducted using adapted and simplified search strings matching search functionality of each website (Additional File 4). The first 100 results from each website were screened in situ. In cases where articles were in a series and published the same data with updates over time, we included the most recent article only.

#### Call for literature

We conducted a call for literature by reaching out to 57 stakeholders, including resource managers, to request gray literature. These calls for literature were sent to experts in the US and US territories (Puerto Rico, US Virgin Islands), Australia, Spain, United Kingdom, France, Monaco, Israel, Saudi Arabia, and several international organizations between September 26 - October 2, 2023. In several instances, stakeholders shared reference libraries (e.g., .bib, .ris) with over 100 references; in these cases, we screened the first 100 results from each reference library in situ.

#### Assembling and managing search results

Search results from the indexing platforms, bibliographic database, open discovery citation indexes, web-based search engine, and novel literature discovery tool were downloaded as .RIS files. All .RIS files were imported into R version 4.2.2 [49], assigned a source (e.g., Web of Science, Scopus, LENS), and deduplicated using CiteSource [50]. The deduplicated references were exported from R as a .RIS file, which was then imported to EndNote version 21.2 [51] for manual deduplication. Deduplication was conducted following steps in McKeown and Mir [52]. Duplicates were merged but the record ID of the discarded duplicate was collated to the record ID of the retained duplicate for tracking. Articles from organizational websites and the call for literature were deduplicated during in situ screening and added to the final map .RIS file.

### Article screening and study eligibility criteria

#### Screening process

Screening was conducted in two stages, first by title and abstract and second by full text.

We used the software Swift-Active Screener (henceforth Swift) [53] for title and abstract screening. Swift uses a combination of screener feedback and a type of machine learning termed “active learning.” The active learning algorithm continuously incorporates screener feedback on which articles should be included or excluded based on the title and abstract screening decisions. The software then ranks the remaining unscreened articles in order of relevance. The most relevant articles are then prioritized for screening. We conducted title and abstract screening in Swift until the software’s “recall rate” reached 95%. The “recall rate” is the running estimate of the percentage of relevant articles that have been screened from the original set.

Four screeners (ABP, CC, DNS, LP) conducted title and abstract screening in Swift. Prior to screening, all screeners attended a training session (led by ABP) that provided background on the project and taught them how to screen and use Swift. During the session, 10 articles were screened together. Following the training session, everyone screened 10 articles independently; we then compared responses and discussed and resolved inconsistencies. The team then conducted a third screening exercise, where everybody screened an additional 30 articles independently, and again compared responses and discussed inconsistencies. Next, we evaluated inter-reviewer consistency on a set of 100 randomly selected articles. We used percent agreement to evaluate consistency, and each pair of reviewers achieved 95% or higher agreement, suggesting that single-screening was sufficient. Screeners were then authorized to begin screening in earnest; if a screener was unsure whether an article should be included or excluded, they marked the article as requiring a second opinion from another screener.

Following the completion of title and abstract screening in Swift, we randomly selected 500 articles using a custom R code to be rescreened for quality assurance and quality control. We manually rescreened the selected 500 articles at the title and abstract level. This number of articles was equivalent to 2.5% of the total number of deduplicated articles from database searches (19,434 articles, including those manually included, manually excluded, and excluded by the active learning algorithm) or 7.5% of the manually screened articles (6,643 articles manually included or excluded). There was one article that was originally excluded during title and abstract screening that we deemed potentially relevant and so changed to include. When we changed this article to include within Swift, the recall rate fell slightly below the 95% threshold, so we screened additional articles until our recall rate returned to ≥ 95%.

Full text screening was conducted in an online spreadsheet (detailed description below) so that our multi-institutional team could simultaneously screen individual articles. Full texts were stored in and accessed via EndNote. Screening was conducted by five screeners (ABP, CC, IF, DNS, LP). All screeners attended a training session (led by ABP) to learn how to conduct full text screening and to practice screening articles together. During full text screening, if a reviewer encountered uncertainties, the reviewer discussed these uncertainties with at least one other reviewer or in some cases the whole team to resolve the problem. Lessons learned from these discussions were noted and provided for the whole team. Following full text screening, we conducted quality assurance and quality control by independently rescreening 25 articles (5%). The 25 articles were randomly selected using a custom R code. This number of articles was equivalent to 5% of the total number of full text articles for which full texts (499) could be retrieved; it did not include articles from organizational websites or stakeholder contributed literature. During rescreening, the full texts were screened independently by a second reviewer. Any inconsistences were noted, discussed, and resolved. There were two articles that required changes to the full text screening decision; one article had a qualitative outcome in the discussion section, and the other had a nursery structure that reported on fish but also coral.

#### Eligibility criteria

Articles were screened using the following eligibility criteria.

- **Relevant population**: The relevant population was coral reefs located in shallow tropical waters. We define shallow as ≤ 30 m. We define tropical waters as those between 35^o^N and 35^o^S latitude; this may include some water typically designated as subtropical depending on the latitudinal classification scheme. Reef types include atolls, fringing reefs, barrier reefs, and generic reefs. If a coral reef was created by a built structure intervention on sand, then it was included. Coral reefs located in deep waters or mesophotic zones were excluded. Reefs with substrate other than carbonate deposited by coral, such as rocky reefs or sponge reefs, were also excluded. All other marine, coastal, terrestrial, freshwater, and subterranean ecosystems were also excluded.
- **Relevant intervention**: Relevant interventions used a built structure, such as those of: (1) Artificial or human-made origin, including structures engineered or designed for reef contexts with or without electricity, structures repurposed from their primary use, and those structures created as artwork; (2) Hybrid origin that are created from a combination of artificial and natural material, such as cement plus natural rock; (3) Natural origin from geologic sources, such as mined rock, limestone, or boulders. These interventions were related to coral reef-related applications, including restoration, remediation, mitigation, enhancement, rehabilitation, rebuilding, stabilization, providing coastal protection or defense, tourism and recreation, research, etc. These interventions were established in response to general habitat degradation and chronic disturbances or in response to pulse disturbances, like storms, blast fishing, dredging, mining, and ship groundings. Interventions using electrification and a built structure were included. Built interventions that were unintentional coral habitat were also included (see refined eligibility criteria below).
- **Relevant comparator**: Studies with comparators over time or space. Comparators included: presence vs. absence of built structure intervention, before vs. after built structure intervention, different types of built structure interventions, different projects or sites with the same built structure intervention type, different reef types (e.g., built structure on fore- vs. back reef), built structure vs. natural coral reef. If articles did not have comparators, however, they could also be included. For instance, if an article measured coral cover on a built structure once but did not compare over space or time, the study contained valuable evidence and was thus eligible for inclusion.
- **Relevant outcome**: Ecological and physical performance outcomes of built structure interventions that are measured, observed, or modeled. Ecological outcomes relate to coral and coral reef metrics, such as recruitment, growth, mortality, condition, rugosity, and cover. Ecological metrics related to biological interactions with coral were included. Physical outcomes relate to waves, currents, erosion, flooding, and other coastal processes. Performance outcomes could be related to the built structure or adjacent areas. For example, ecological outcomes like coral growing on the built structure or coral growing adjacent to the built structure were both be included.
- **Relevant study type**: Experimental, modeling (statistical, theoretical, simulation), or observational studies with quantitative data. Field and lab studies were included. Reviews, meta-analyses, theoretical studies, commentaries, editorials, opinions, and perspectives were excluded. If lab studies occurred, the country where the laboratory was located could fall outside of the 35^o^N and 35^o^S latitude range.

Several special cases arose for which we refined eligibility criteria. For example, if nursery corals were outplanted onto a bare substrate or dead corals, this was excluded because the substrate was not considered built unless it was purposely placed. If a study examined coral recruitment on natural substrate but used settlement tiles to do so, it was excluded because the settlement tiles were used to measure natural recruitment rather than effects from a built structure; if settlement tiles were used on a built structure, however, they were included. We also refined our inclusion criteria to encompass interventions that were unintentional coral habitat. Unintentional built structures included accidental or historic shipwrecks. The unintentional category also included built structures, such as aquaculture infrastructure, that were not designed for coral reef-related applications but formed *de facto* coral habitat. Other unintentional examples that were included were seawalls or jetties not originally intended for restoration or other reef-related applications but that did contain measurements of coral growth, coral settlement, or other coral metrics. Additionally, if a study examined the performance of a built structure in a laboratory environment but did not feature coral, then it was excluded.

#### Study validity assessment

Study validity was not systematically assessed because this systematic map aimed to collate and summarize the distribution and abundance of evidence. During data coding, however, attributes were extracted that can be used for follow-up assessments of study validity for subsets of the evidence base.

#### Data coding strategy

Metadata from studies that passed full text screening were entered into a data “coding” spreadsheet. Each study corresponded to one row in the spreadsheet. These attributes included bibliographic information, as well as those related to the population, intervention, study type, comparator, and outcome (Additional File 5) and associated typologies (Additional File 6). Details of each attribute were provided in a code book that describes each attribute, instructions for data entry, and levels of categorical attributes that screeners could select from dropdown menus (Additional File 5). Data were coded according to information in the full text and supplementary materials; we did not contact authors to request missing information.

Three researchers (ABP, CC, DNS) piloted the codebook on 10 articles and discussed challenges and inconsistencies. The five data coders (ABP, CC, DNS, IF, LP) were trained on metadata extraction during the full text training session (led by ABP). We did not conduct double extraction because of the high number of articles that required coding. Instead, we discussed articles that were unclear or challenging. The coding tool was deployed in an online spreadsheet. After completing data coding, spot checks were conducted for 100% of the included coded articles discovered during the full database searches, call for literature, and organizational website searches. During spot checks, we checked for and corrected dissimilarities in spelling, deviations from pre-defined factor levels, ambiguous metadata, and any other uncertainties. Following spot checking, coded data were exported from the online spreadsheet as a .csv and imported to R for analysis and visualization.

#### Data mapping method

Coded data were analyzed in R version 4.2.2 [49] to answer the primary and secondary research questions. We provided an overview of the review process using the ROSES flow diagram [54]. We then visualized the evidence base by descriptive information (e.g., publication type, publication date, geography), coral reef types, seascape settings, built structure intervention types, study types, and ecological and physical outcomes. We also compiled heatmaps where ecological and physical outcomes (rows) were mapped against built structure intervention types (columns) to assess evidence clusters and evidence gaps. All visualizations were created using ggplot2 [55].

## Review findings

### Systematic mapping process

**Fig. 1:**
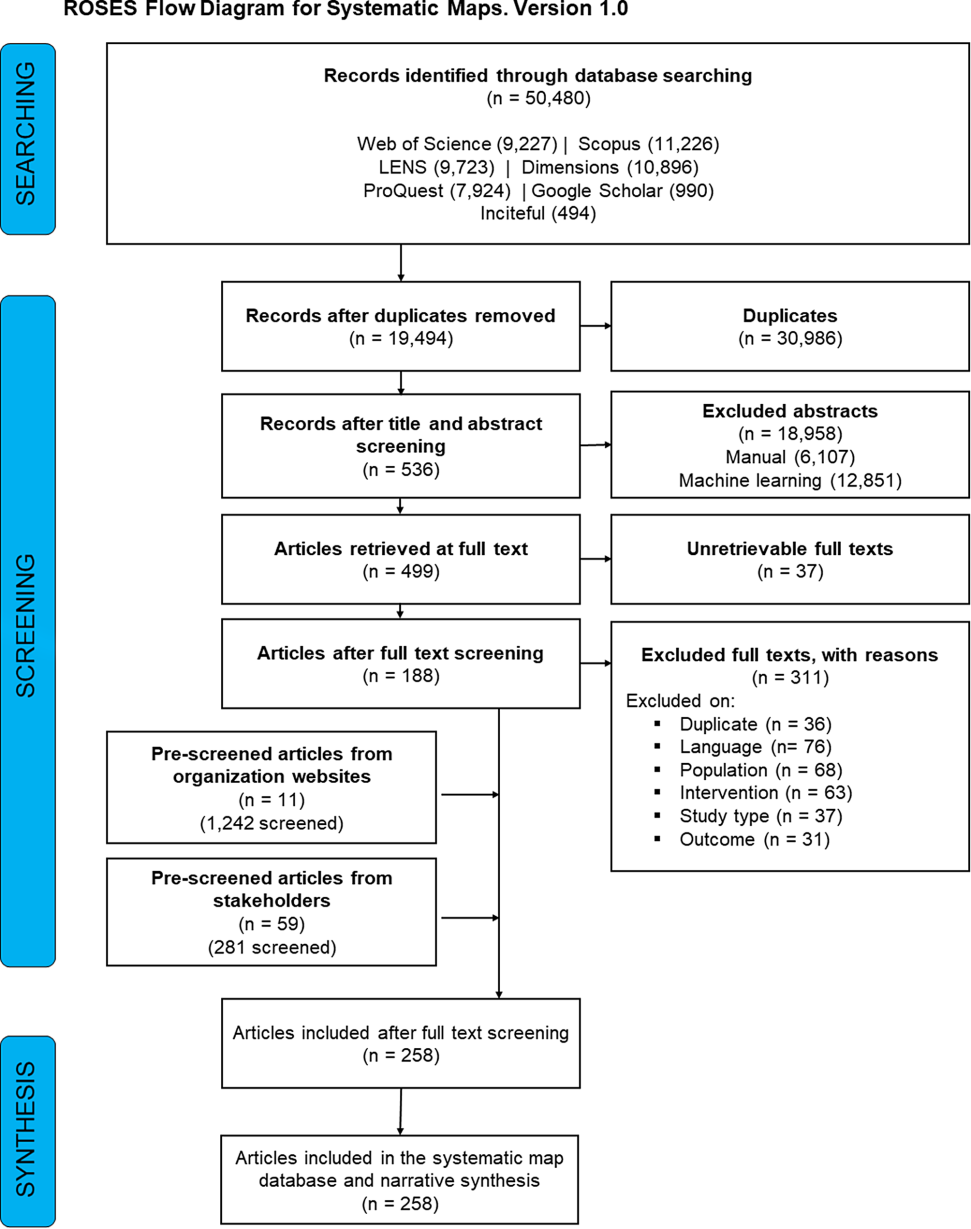
ROSES flowchart depicting the number of articles returned from the initial search and included during each stage in the map process. Flowchart from Haddaway, Macura, Whaley and Pullin [54]

The number of articles returned during each stage in the systematic map process is reported in the ROSES flowchart (Fig. 1). Database searches returned 50,480 potentially relevant records. Scopus returned the highest number of records (*n* = 11,226), followed by Dimensions (*n* = 10,896), LENS (*n* = 9,723), Web of Science (*n* = 9,227), and ProQuest (*n* = 7,924). The web-based search engine Google Scholar yielded 990 records, and the novel search tool Inciteful [48] discovered 494 records. After deduplicating articles across the databases, 19,494 articles remained (*n* = 30,986 duplicates) and were screened at the level of title and abstract. During title and abstract screening, 536 articles were included, and a high proportion of articles were excluded (*n* = 18,958) either by manual screening (*n* = 6,107) or machine learning (*n* = 12,851) using Swift Active Screener [53]. Of the 536 articles that passed title and abstract screening, full texts were retrievable for 499 (*n* = 37 unretrievable; Additional File 8). During full text screening, 188 articles were included and 311 were excluded. Articles were excluded during full text screening because they were not in English (*n* = 76), did not meet our criteria for a coral reef population (*n* = 68), did not have a built structure intervention (*n* = 63), were the improper study type (*n* = 37), were duplicates that had been missed at the previous step or reported on the same content using a slightly different title or format (e.g., report vs. journal article) (*n* = 36), or did not have eligible ecological or physical outcome (*n* = 31).

Outside of database searches, articles were sourced from organizational website searches and a call for stakeholder contributed literature. Organizational website searches yielded 1,242 potentially relevant articles; 11 articles were included based on in situ screening. Calls for literature contributions from stakeholders retuned 281 potentially relevant articles, of which 59 were included based on in situ screening.

In total, 258 articles (*n* = 188 from databases, *n* = 11 from organizational websites, and *n* = 59 from stakeholder-contributed literature; full bibliography Additional File 7) were included in the systematic map after full text screening; these articles are included in the systematic map database and narrative synthesis. The bibliography of excluded articles and their exclusion reasons is in Additional File 8. Coded data for included articles is in Additional File 9. The ROSES reporting form is in Additional File 1. In the descriptive results reported below on publication information, reef type, seascape setting, intervention types, study types, and outcomes, articles can appear in more than one category (e.g., an article can contribute to the sample size for multiple categories, so the total sample size can be greater than the number of total articles (258)).

### Descriptive information

#### Publication type

**Fig. 2:**
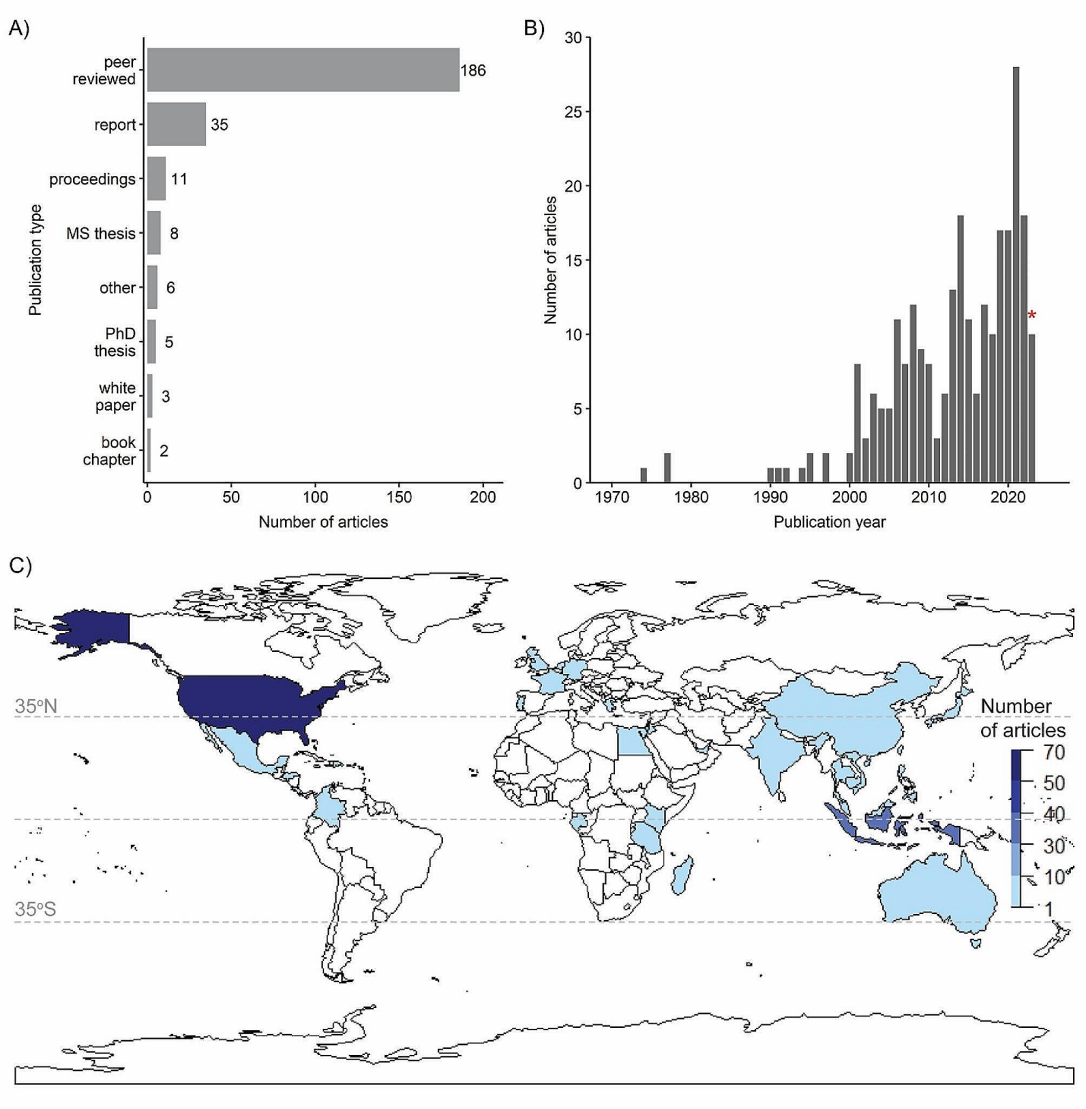
Number of articles by (**A**) publication type, (**B**) publication year, and (**C**) country. For (**B**), Red asterisk in panel B denotes a partial year for 2023, which is when the search was conducted. For (**C**), countries that are not in blue denote 0 articles. Dashed lines indicate 35°N to 35°S latitudes, as well as the equator. Some European countries that are outside of the 35°N to 35°S latitudinal range contain evidence either because *ex-situ* studies were conducted in these countries or because *in-situ* studies occurred in associated locations, such as Little Cayman for the United Kingdom; see Table 1 for additional details

The majority (72.1%) of articles in the map were peer-reviewed publications (*n* = 186; Fig. 2A). Reports comprised 13.6% (*n* = 35) of the evidence base and proceedings 4.3% (*n* = 11). The remaining 10.0% were several MS theses, PhD dissertations, white papers, and book chapters.

**Table 1:** Geographic distribution of evidence by country where the research was conducted

Country	Number of articles	Country	Number of articles
United States	68	Qatar	2
Indonesia	30	United Kingdom^**1**^	2
Israel	18	Antigua and Barbuda	1
Singapore	12	Bahamas	1
Australia	10	Barbados	1
Philippines	10	Brunei Darussalam	1
Japan	9	Cambodia	1
Maldives	9	Costa Rica	1
Malaysia	8	France^**2**^	1
Thailand	8	French Polynesia	2
India	6	Gabon	1
Egypt	5	Germany^**3**^	1
Jordan	5	Greece	1
Mexico	5	Grenada	1
Netherland Antilles	5	Guadeloupe	1
Puerto Rico	4	Honduras	1
Vietnam	4	Jamaica	1
China	3	Madagascar	1
United Arab Emirates	3	Marshall Islands	1
Colombia	2	Mauritius	1
Curacao	2	Palau	1
Dominican Republic	2	Portugal^**4**^	1
Fiji	2	Seychelles	1
Kenya	2	Taiwan	1
Kuwait	2	Tanzania	1

Details of geographic locations of articles from several countries:

^1^United Kingdom: Turks and Caicos (*n* = 1), Little Cayman (*n* = 1)

^2^France: New Caledonia (*n* = 1)

^3^Germany: Ex-situ setting (*n* = 1)

^4^Portugal: Ex-situ setting (*n* = 1)

#### Publication year

The number of articles published per year increased over the past five decades (Fig. 2B); the earliest published article was from 1974. There were 3 articles published in the 1970s, zero in the 1980s, and 8 in the 1990s. At the turn of the century, the number of published articles grew, totaling 69 in the 2000s and 104 in the 2010s; there have been 73 publications so far in the 2020s. The two most recent years, 2021 and 2022, had the highest annual number of publications to date, 28 and 18, respectively. The year 2023 is incomplete because the search ended before the end of the calendar year; database searches were completed in April 2023 and organizational websites were searched in September 2023.

#### Publication geography

The evidence base stemmed from 50 countries (Fig. 2C; Table 1). The countries where the research occurred with the most articles were the United States (*n* = 68), Indonesia (*n* = 30), Israel (*n* = 18), Singapore (*n* = 12), Australia (*n* = 10), and Philippines (*n* = 10). These top six countries represent ∼ 56% of the total evidence base.

### Coral reef types examined

**Fig. 3:**
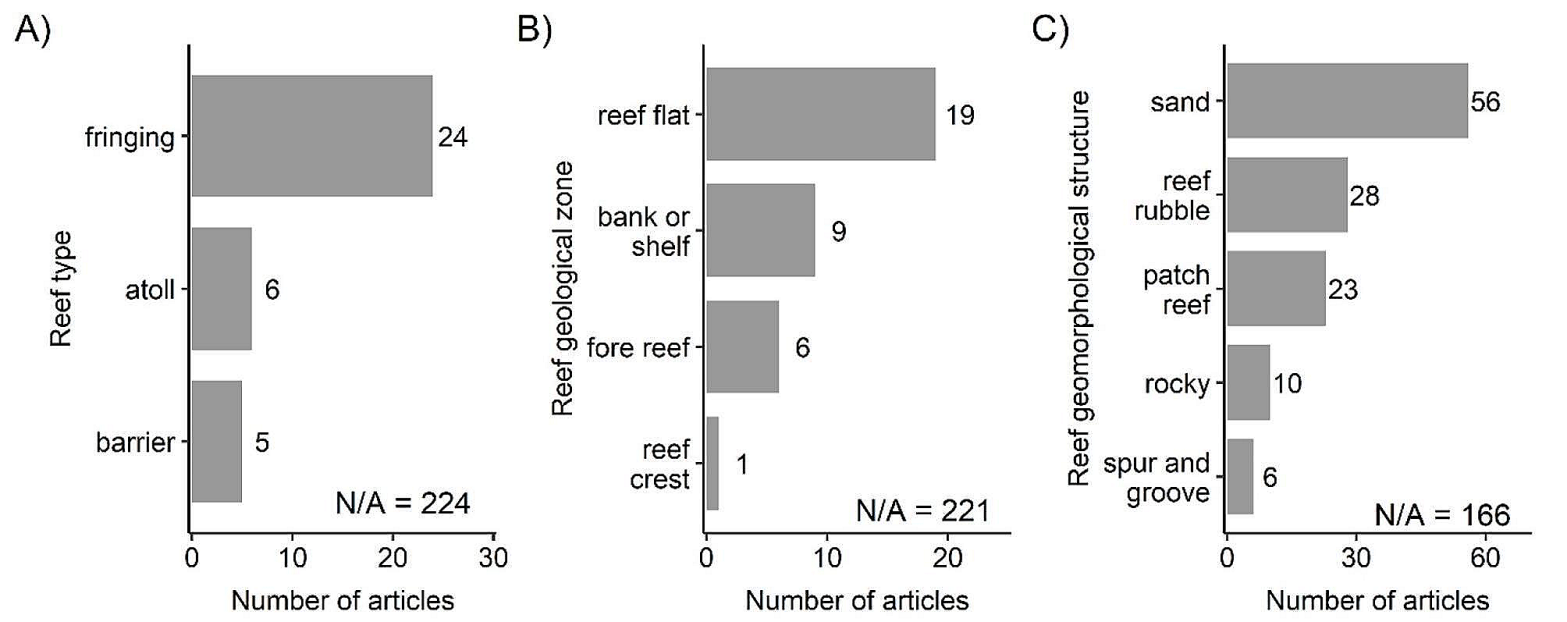
Number of articles by coral reef population characteristics: (**A**) reef type, (**B**) reef geological zone, (**C**) reef morphological structure. Some articles contained more than one population, so articles can appear in more than one category within each panel. “N/A” indicates the number of articles where the reef characteristic was unspecified; for reef type (A), sand is included within “N/A”

Coral reefs populations were classified by reef type, reef geological zone, and reef geomorphological zone according to a USGS and NOAA coral reef classification scheme based on Coyne, et al. [56], Kendall, et al. [57], and Cochran et al. [58]. The majority of articles did not report reef type (*n* = 224), geological zone (*n* = 221), or geomorphological zone (*n* = 166) (Fig. 3). Of the articles that did specify, most evidence stemmed from reef types classified as fringing reefs (*n* = 24) (Fig. 3A) and geological zones classified as reef flats (*n* = 19) (Fig. 3B). The geomorphological structures were commonly sand (*n* = 56), reef rubble (*n* = 28), or patch reef (*n* = 23) (Fig. 3C).

### Seascape settings of studies

**Fig. 4:**
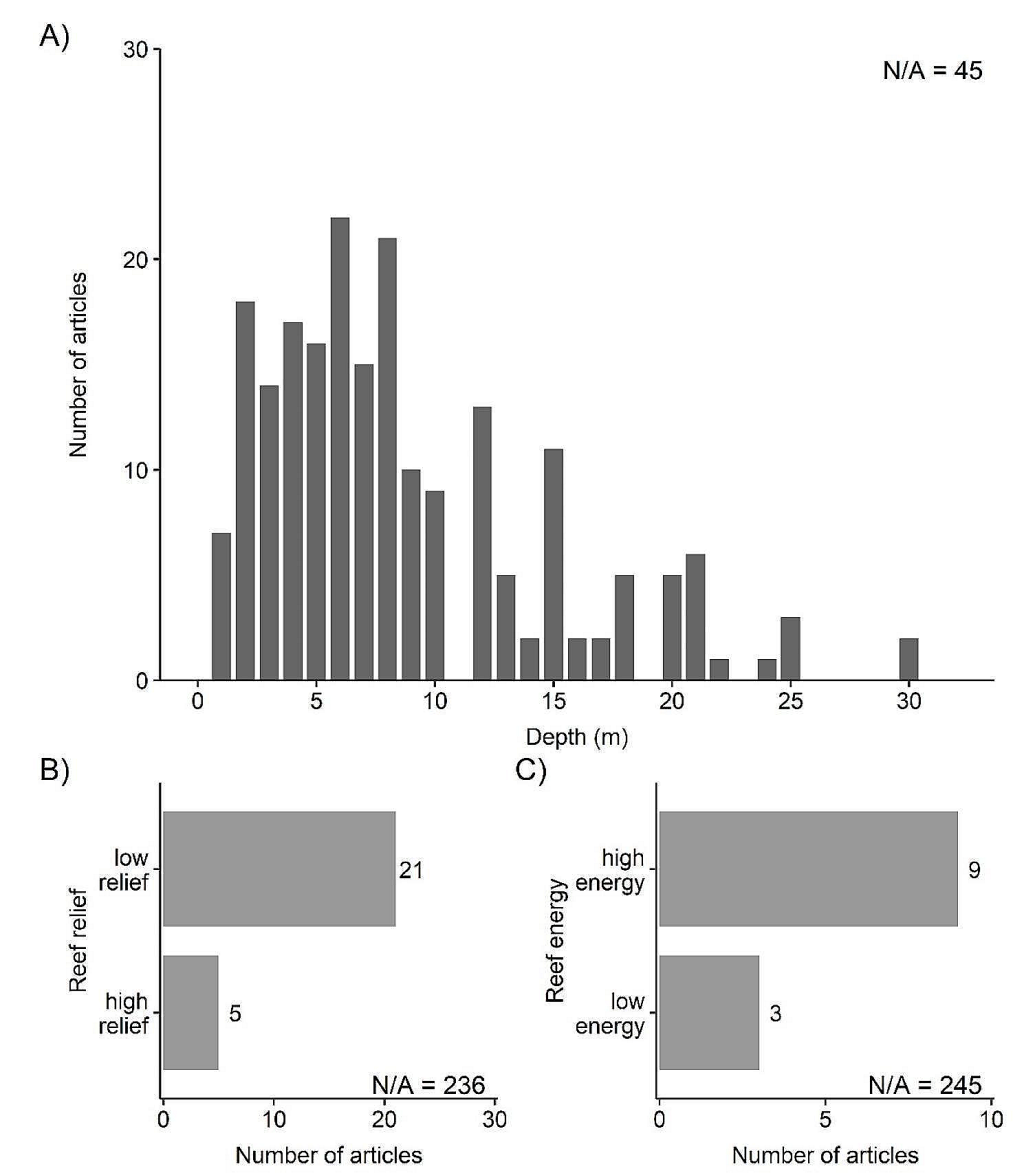
Number of articles by seascape setting: (**A**) depth (m), (**B**) reef vertical relief, and (**C**) reef energy regime. Some articles contained more than one population, so articles can appear in more than one category within each panel. If a study reported more than one depth or depth range, then depths were averaged. “N/A” indicates the number of articles where the seascape setting was unspecified

The seascape setting of the coral reefs and their built structure interventions included the water depth, reef vertical relief, and reef energy regime (Fig. 4). The majority of articles reported on reefs that were at depths ≤ 10 m (*n* = 155; Fig. 4A). There were 45 articles on reefs 11–20 m deep, and 13 articles on reefs 21–30 m deep (Fig. 4A); we selected three equally divided depth bins based on our predefined maximum eligible reef depth of ≤ 30 m. Most studies did not report either the reef vertical relief (*n* = 236) or reef energy regime (*n* = 245). Of studies that did report the relief and energy regime, more occurred on low relief (*n* = 21) than high relief reefs (*n* = 5) (Fig. 4B); more studies also took place on high energy reefs (*n* = 9) than low energy reefs (*n* = 3) (Fig. 4C). The reef relief and reef energy categories were assigned based on qualitative descriptors in the articles.

### Characteristics of built structure interventions

**Fig. 5:**
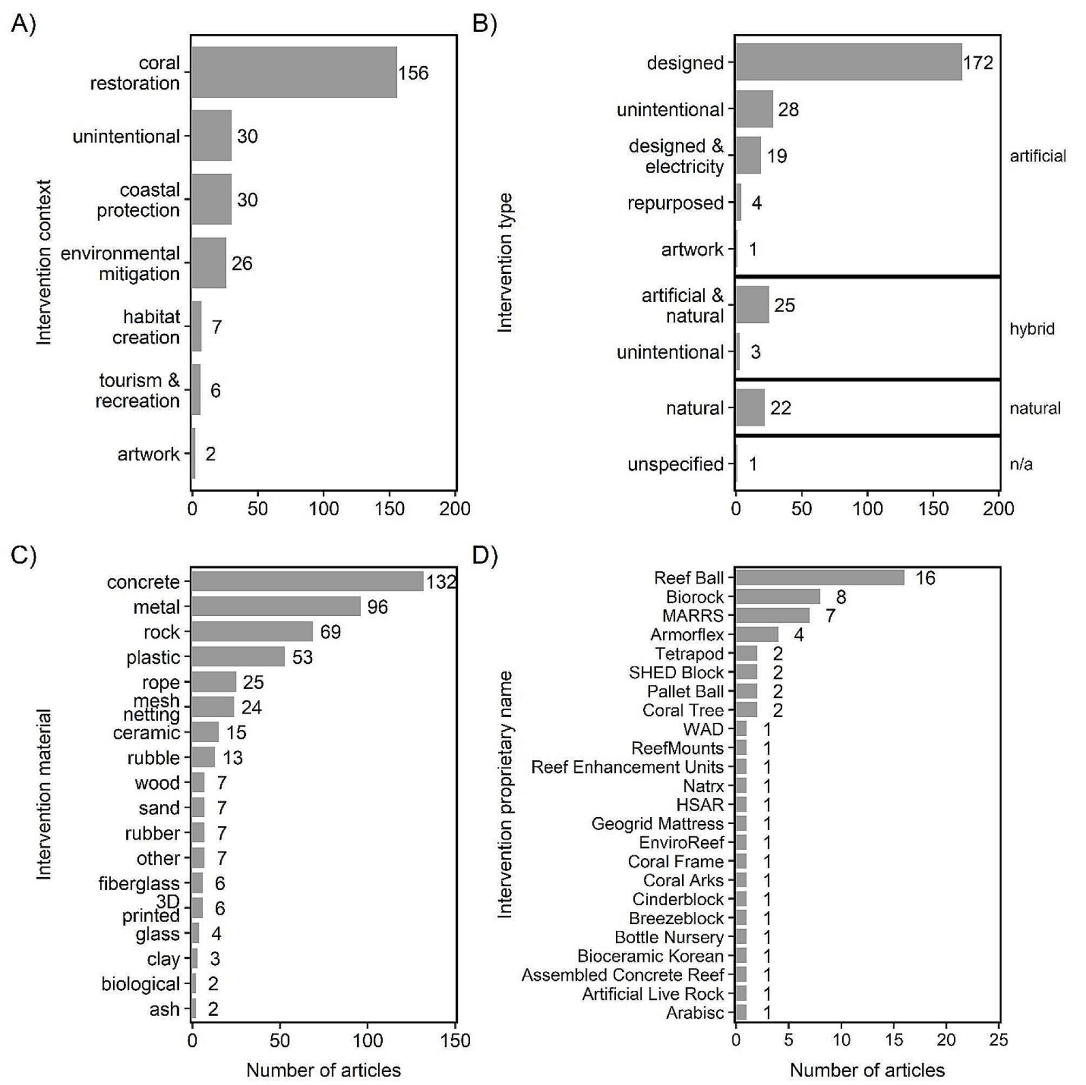
Number of articles by built structure intervention characteristics: (**A**) context, (**B**) type, (**C**) material, and (**D**) proprietary name. Some articles contained more than one built structure intervention, so articles can appear in more than one category within each panel. “N/A” indicates the number of articles where the intervention characteristic was unspecified. Abbreviations for the proprietary names are defined as: HSAR = Hemispherical Shape Artificial Reefs, WAD = Wave Attenuation Device, SHED = Sheppard Hill Energy Dissipator, MARRS = Mars Assisted Reef Restoration System

Built structure interventions spanned a variety of goals, structure types, structure materials, and proprietary structure names (Fig. 5).

#### Context

Most of the interventions were installed for coral restoration (*n* = 156; 60.7%; Fig. 5A). Another common intervention goal was to achieve coastal protection (*n* = 30; 11.7%) against waves energy and sediment changes. Other interventions were unintentional and so had no specific goal (*n* = 30; e.g., ship sunk accidentally), whereas some were installed for environmental mitigation (*n* = 26), habitat creation (*n* = 7), tourism and recreation (*n* = 6), or artwork (*n* = 2).

#### Type

Built structures were classified as either artificial (*n* = 224), hybrid (*n* = 28), or natural (*n* = 22) (Fig. 5B). Of the artificial structures, most were designed (*n* = 172), but some were unintentional so lacked a priori design for reef-related applications (*n* = 28), such as historic shipwrecks and aquaculture infrastructure; others were designed and supplemented with electricity (*n* = 19; e.g., mineral accretion technology). Hybrid structures were mainly designed (*n* = 25), but several (*n* = 3) [59–61] were unintentional. One structure was called an artificial reef, but it was unclear whether it was artificial, hybrid, or natural in origin (*n* = 1 for unspecified – n/a).

#### Material

The most common built structure materials were concrete (*n* = 132), followed by metal (*n* = 96), rock (*n* = 69), and plastic (*n* = 53) (Fig. 5C). Natural built structures were rocks and boulders, primarily composed of limestone.

#### Proprietary name

Some of the built structures were proprietary, such as Reef Balls (*n* = 16), Biorock (*n* = 8), and MARRS Reef Spiders (*n* = 7) (Fig. 5D).

### Study types

**Fig. 6:**
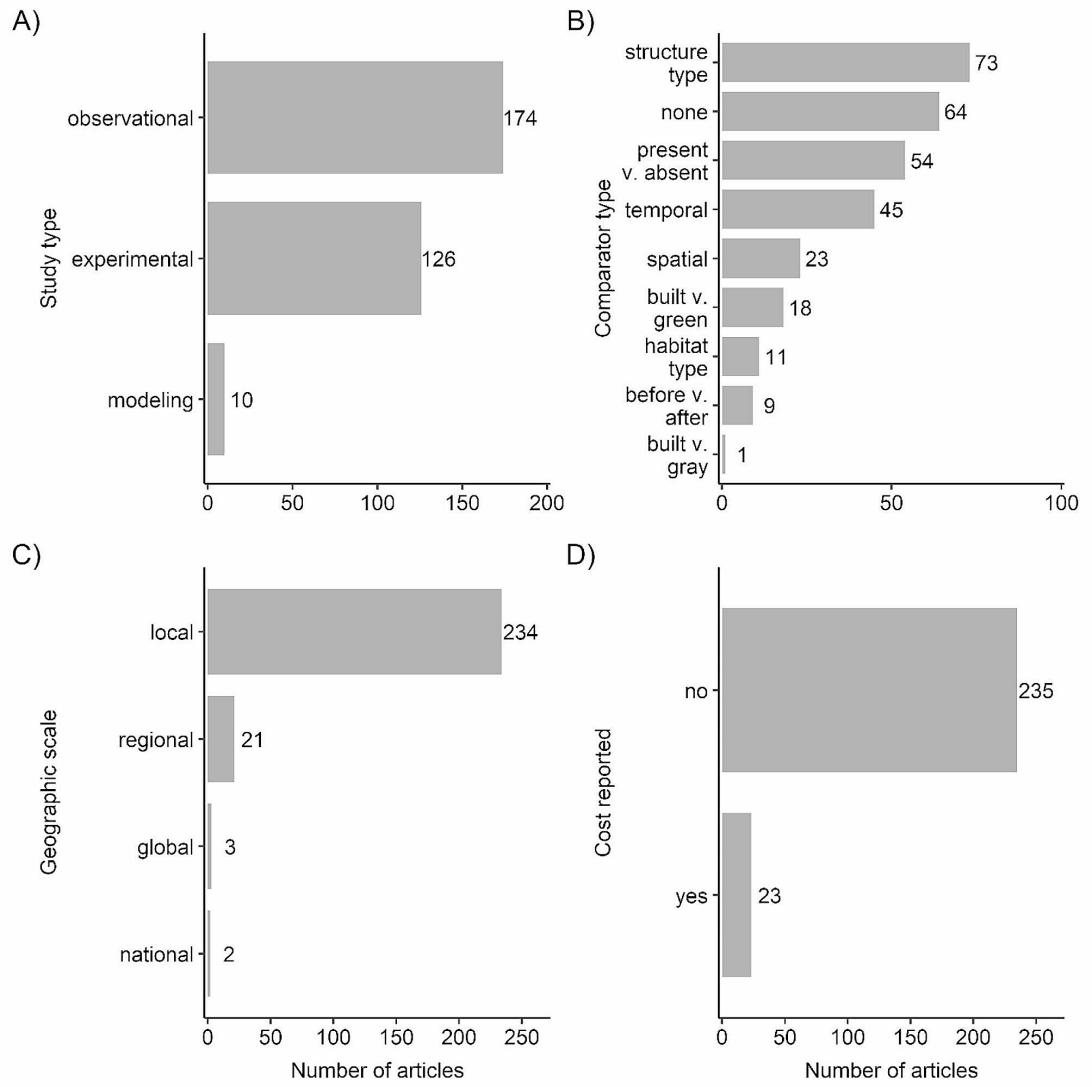
Number of articles by for (**A**) study type, (**B**) study comparator type, (**C**) study geographic scale, and (**D**) whether study reported cost. Some articles contained more than one study type or comparator, so articles can appear in more than one category within each panel. Comparators are: “structure type” compares different types of built structure interventions, “presence v. absence” compares the presence or absence of built structure; “temporal” tracks built structures over time, “none” is no comparator, “spatial” compares built structures at different sites or from different projects, “built v. green” compares a built structure intervention to a green or natural structure, “before v. after” compares before built structure construction to after, “habitat type” compares built structures in different habitat types, “built v. gray” compares a built structure to a fully gray intervention

The evidence base contained a high number of observational studies (*n* = 174) and experimental studies (*n* = 126). Modeling or simulation studies were less common (*n* = 10) (Fig. 6A). Some studies did not have comparators (*n* = 64; Fig. 6B). Of the studies with comparators, most compared different types of built structure interventions to each other (*n* = 73), the presence or absence of built structures (*n* = 54), or tracked built structures over time (*n* = 45). Other studies compared the same types of built structures across spatial scales (*n* = 23), built structures to natural or green systems (*n* = 18, e.g., reef module versus natural coral reef), built structures in different habitat types (*n* = 11; e.g., reef modules in high energy environment vs. low energy environment or reef modules in urban versus natural settings), before versus after built structures (*n* = 9), and built versus fully gray infrastructure (*n* = 1; e.g., reef module versus bulkhead not intended for coral reef-related applications). The geographic scale of the studies was frequently local (*n* = 234), although there were several regional (*n* = 21), global (*n* = 3), and national (*n* = 2) studies (Fig. 6C). Only 23 studies or 8.9% reported the cost of the built structure intervention (Fig. 6D).

### Ecological and physical performance outcomes examined

**Fig. 7:**
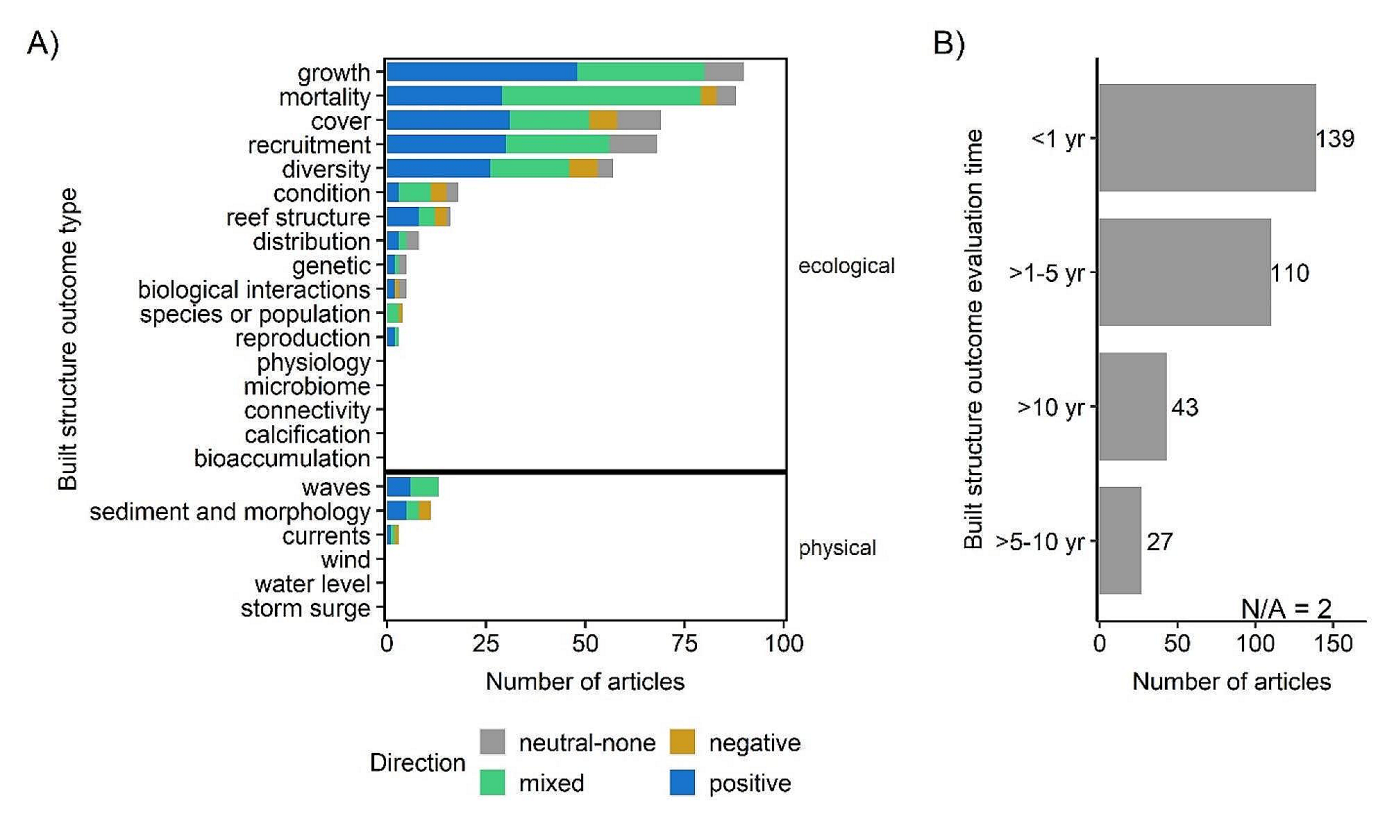
Number of articles by (**A**) outcome category and (**B**) outcome evaluation time. Outcome types for coral ecological outcomes and physical outcomes (**A**) are colored by the outcome directionality and faceted by whether the outcome is ecological (top panel) or physical (bottom panel). Outcome directionality (e.g., positive and negative) does not infer statistical significance. Outcome evaluation times (**B**) are relative to built structure construction, where time periods are the number of years following construction. Some articles contained more than one outcome, so articles can appear in more than one category within each panel. “N/A” indicates the number of articles where the outcome evaluation time period was unspecified

More articles examined the coral ecological performance outcomes of built structures (*n* = 431) than physical performance outcomes (*n* = 27). The most frequently studied coral ecological outcomes were coral growth (*n* = 90), coral mortality (*n* = 88), coral cover (*n* = 69), coral recruitment (*n* = 68), and coral diversity (*n* = 57; Fig. 7A). Several coral ecological outcomes – physiology, microbiome, connectivity, calcification, bioaccumulation – were not represented in the evidence base. Only three types of physical performance outcomes were studied – those related to waves (*n* = 13), sediment and morphology (*n* = 11), and currents (*n* = 3). Wind, water level, and storm surge were not assessed in the identified evidence base.

Performance outcomes were evaluated using multiple metrics common to ecology and to physical sciences (Table 2). Popular metrics used to quantify ecological diversity, for example, included species diversity, species evenness, species richness, and community composition. For currents, current speed and magnitude were commonly evaluated physical metrics. The methods used to evaluate ecological outcomes often relied upon in situ visual transects, photograph surveys, and video surveys (Table 3). Several studies used settlement tiles and microscope analysis. Some studies employed less common evaluation methods like habitat mapping and eDNA sampling.

**Table 2:** Common metrics for (A) ecological coral outcomes and (B) physical outcomes associated with built structures

Category	Subcategory	Metric
A) Ecological	Coral mortality	Percent mortality (or survival)
		Larval mortality (or survival)
		Transplant mortality (or survival)
		Percent detached
		Cause of death
		Mortality count (or survival)
	Coral growth	Growth rate (height, width, diameter)
		Coral size (height, width, length, diameter, volume, weight)
		Fragment size (height, width, length, diameter)
		Colony size (heigh, width, length, diameter)
		linear extension
	Coral cover	Coral cover
		Percent cover
	Coral recruitment	Number of settlers or recruits
		Settlement behavior
		Recruitment density
		Recruitment percent
	Coral diversity	Species diversity
		Species evenness
		Species richness
		Community composition
	Coral condition	Bleaching proportion
		Bleaching frequency
		Bleaching severity
		Coral condition
		Percent corals per health category
		Tissue stress proportion
	Reef structure	Structural complexity
		Reef height
		Reef size
	Coral distribution	Spatial distribution
	Coral species or population	Abundance
		Density
		Number of functional groups
	Coral biological interactions	Interactions with reef fish
		Interactions with macroalgae
		Interactions with macroinvertebrates
	Coral reproduction	Number of gonads
		Number of settlers or recruits
		Size threshold of fecund corals
	Coral genetic	Genetic diversity
		Genetic abundance
		Genetic richness
		Gene expression
B) Physical	Waves	Wave attenuation
		Wave energy
		Wave height
	Sediment and morphology	Shoreline morphology
		Elevation change
		Sedimentation rate
	Currents	Current speed
		Current magnitude

**Table 3:** Common evaluation methods for (A) ecological coral outcomes and (B) physical outcomes associated with built structures

Category	Subcategory	Method
A) Ecological	Biological interactions	Video surveys
		In situ visual transect
	Coral condition	In situ visual transect
		Photograph survey
		Mark and monitor
	Coral cover	In situ visual transect
		Photograph survey
		Video surveys
		In situ quadrat
		Photogrammetry
		Settlement tile
	Coral distribution	In situ visual transect
		Photograph survey
		In situ quadrat
		Habitat mapping
	Coral diversity	In situ visual transect
		In situ quadrat
		Microscope
		Photograph survey
		Photogrammetry
		Video surveys
		Edna sampling
	Coral genetic	Genotyping
		RNA extraction and analysis
		Amplified fragment length polymorphism analysis
	Coral growth	In situ measurement
		Photograph survey
		Video surveys
		In situ visual survey
		Mark and monitor
		Stereo microscope
		Dissecting microscope
	Coral mortality	In situ measurement
		Photograph survey
		Video surveys
		Settlement tile
		Photogrammetry
		Model estimate
		Dissecting microscope
		Mark and monitor
	Coral recruitment	Settlement tile
		In situ visual transect
		In situ quadrat
		Photograph survey
		Video surveys
		Dissecting microscope
		Stereo microscope
		In situ measurement
		Blue light fluorescence survey
		Hydrodynamic model
		Mark and monitor
	Coral reef structure	In situ measurement
		In situ visual survey
		Rugosity (chain method)
	Coral reproduction	Coral branch surveys
		Larval settlement survey
	Coral species or population	Photogrammetry
		Remotely-operated vehicle (video)
		In situ visual transect
	Coral survival	In situ visual survey
B) Physical	Currents	Clod card
		Gypsum block
		Eddy simulation
		Dye tracking
	Sediment	Sediment traps
		Clod card
		Elevation remote sensing
	Waves	Hydrodynamic model
		Wave gauge
		Wave flume

Most articles reported on outcome evaluations within a year of construction (*n* = 139) or up to five years following construction (*n* = 110; Fig. 7B). Fewer articles presented longer-term outcome evaluations up to 10 years post construction (*n* = 27) or more than 10 years post construction (*n* = 43). Outcome evaluations indicate that the directionality of evidence (e.g., positive, negative, neutral, or mixed) varied (Fig. 7A). In some cases, the performance of the structure was positive, whereas in others it was negative, neutral, or mixed (both positives and negatives reported). For instance, if there were enhanced ecological outcomes (e.g., increased growth, decreased mortality, increased diversity), then the directionality of evidence was coded as “positive.” Following this coding approach, evidence related to coral mortality was mixed in 50 cases, positive in 29, negative in 4, and neutral in 5. Likewise, if there were enhanced physical outcomes (e.g., reduced wave attenuation, reduced erosion) then the outcome was coded as “positive.” For waves, evidence was mixed in 7 instances and positive in 6.

### Intersection of built structure interventions and ecological performance outcomes

**Fig. 8:**
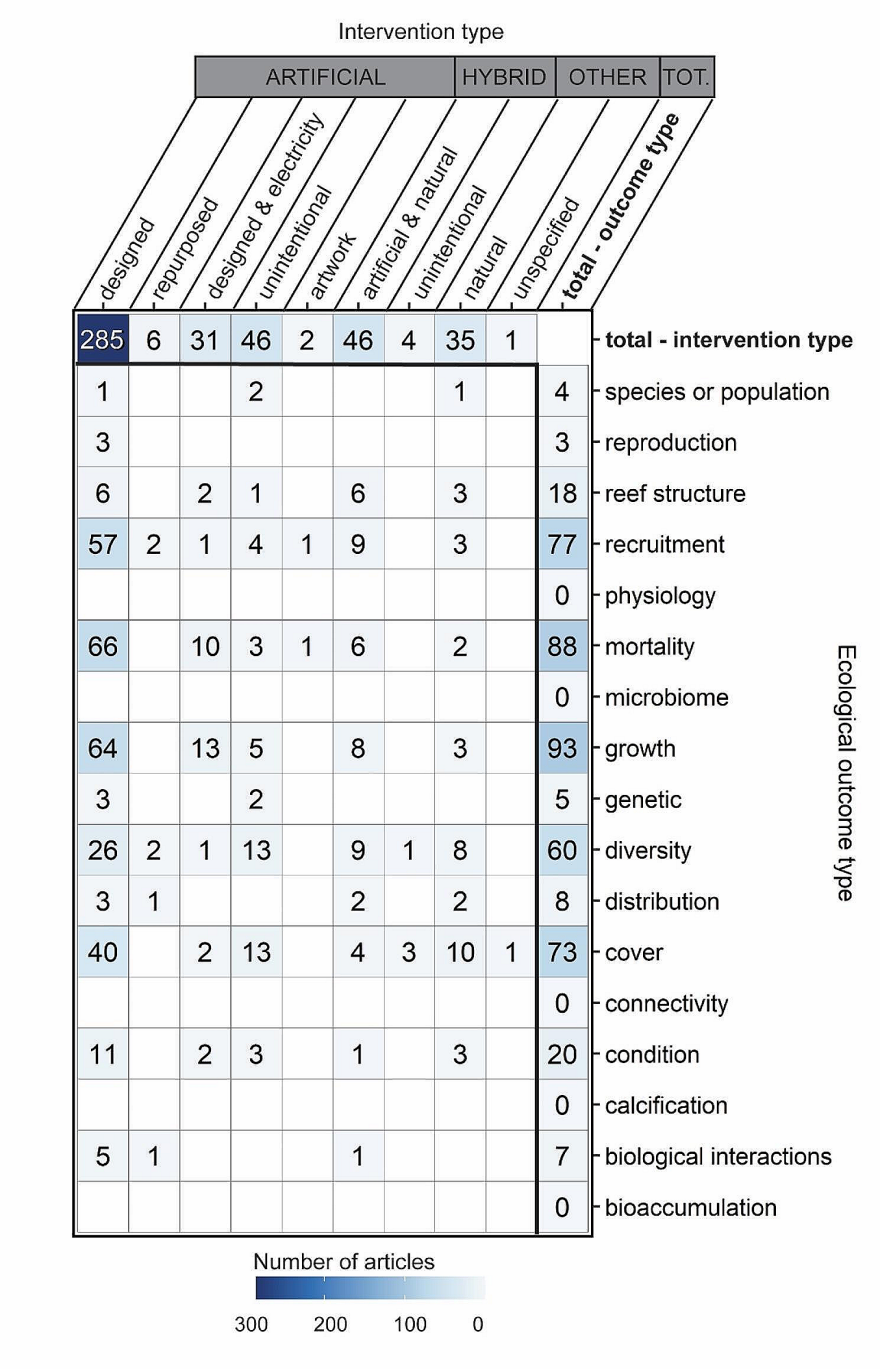
Distribution of evidence (number of articles) across built structure intervention types and coral ecological outcomes. Some articles contained more than one intervention or outcome, so articles can appear in more than one cell. Blank cells have zero articles. Top row and far right column provide total values across intervention types and coral ecological outcomes, respectively

Evidence clusters were most pronounced for coral ecological performance outcomes on artificial structures that were designed (without electricity or mineral accretion technology) for reef-related applications (Fig. 8). For example, the largest evidence clusters on artificial designed structures were for coral mortality (*n* = 66), growth (*n* = 64), recruitment (*n* = 57), cover (*n* = 40), and diversity (*n* = 26). Artificial structures that were designed with electricity had a moderate amount of evidence for coral growth (*n* = 13) and mortality (*n* = 10); artificial unintentional structures had a moderate amount of evidence for cover (*n* = 13) and diversity (*n* = 13). Hybrid structures had a moderate amount of evidence for diversity (*n* = 9), recruitment (*n* = 9), and growth (*n* = 8). The most pronounced gaps were for particular outcomes (e.g., connectivity, physiology, bioaccumulation, calcification) without evidence across all built structure types, as well as for artificial artwork (*n* = 2 outcomes), artificial repurposed (*n* = 6 outcomes), and hybrid unintentional (*n* = 4) outcomes.

### Intersection of built structure interventions and physical performance outcomes

**Fig. 9:**
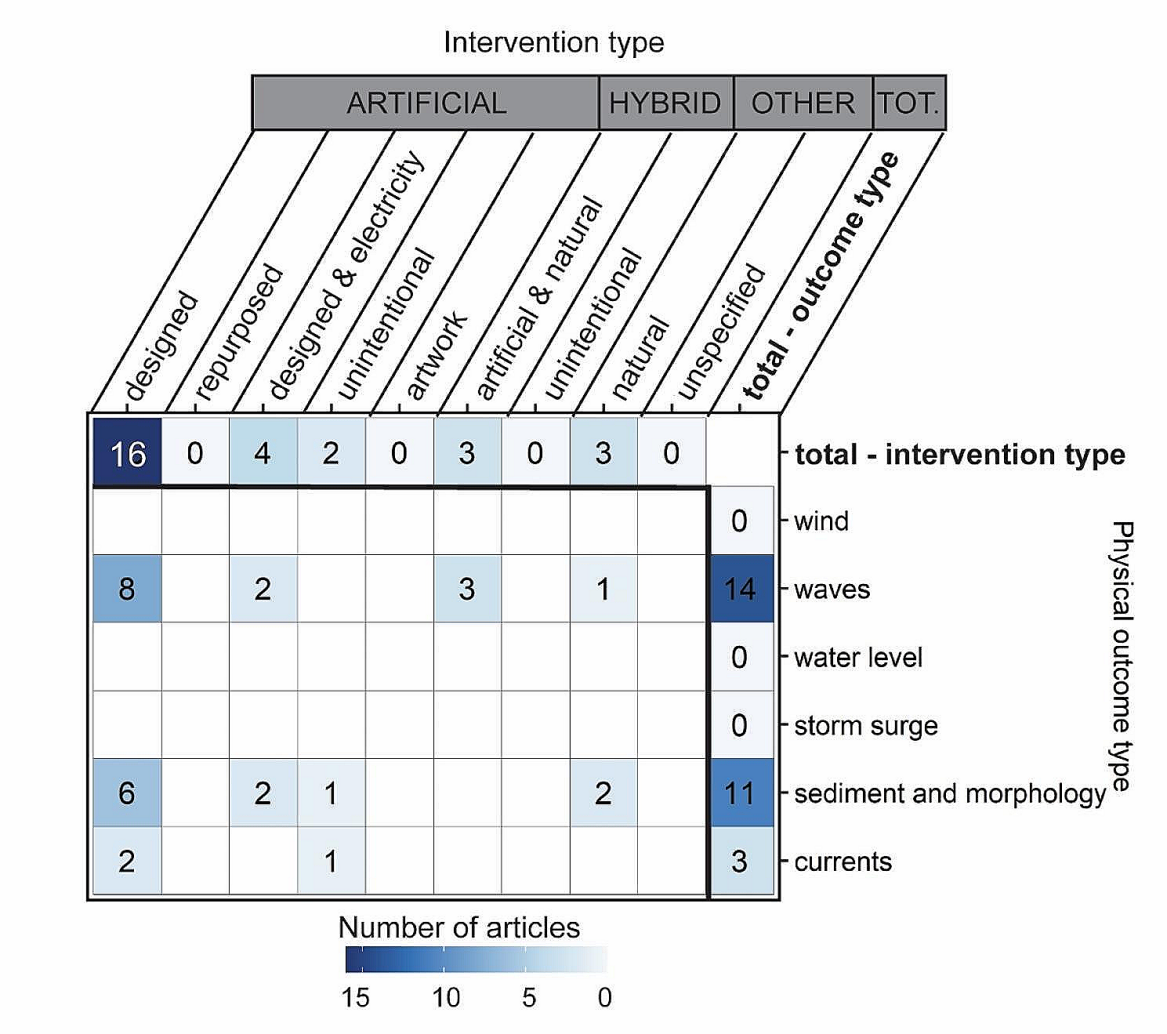
Distribution of evidence (number of articles) across built structure intervention types and physical outcomes. Some articles contained more than one intervention or outcome, so articles can appear in more than one cell. Blank cells have zero articles. Top row and far right column provide total values across intervention types and physical outcomes, respectively

Evidence on the physical performance of built structures was relatively sparse (Fig. 9). Most evidence stemmed from outcomes related to waves (*n* = 8) and sediment and morphology (*n* = 6) on artificial built structures designed for reef-related applications. There was sparse evidence related to currents (*n* = 2) on artificial built structures, as well as for wave outcomes (*n* = 3) on hybrid structures. Unintentional artificial built structures were rarely evaluated for sediment and morphology (*n* = 1) and current (*n* = 1) outcomes, as were artificial structures designed with electricity (waves *n* = 2, sediment and morphology *n* = 2). Similarly, natural structures were rarely evaluated for waves (*n* = 1) and sediment and morphology (*n* = 2). Complete gaps in evidence exist at all other intersections of built intervention types and physical outcomes.

### Evidence clusters and gaps

The systematic map provides an up-to-date published evidence base on the ecological (coral-related) and physical performance of built structures for reef-related applications in shallow coral ecosystems through 2022 and partially through 2023, when the search was conducted. Map findings highlight the distribution and abundance of evidence by publication type, year, and country, as well as characteristics of the coral reef population, built structure intervention, comparator, and outcomes. Taken together, our findings highlight several evidence clusters related to the ecological performance of designed artificial structures for coral, but also a multitude of evidence gaps. Evidence gaps were most pronounced across physical outcomes on all types of built structures, as well as for some ecological outcomes especially on artwork and repurposed structures. The compiled evidence base can help guide manager consideration of whether and how to incorporate built structures into coral-reef related applications, such as restoration, coastal protection, and environmental mitigation. Here, we discuss implications of our findings on the evidence base related to the built structure goal, structure type, and materials. We then highlight the ecological and physical gaps and limitations of the map.

#### Goal of built structures

The abundance of evidence differed according to the intervention goal. Most built structures were installed to meet coral restoration goals. These goals largely match those reported for broader coral restoration by Bayraktarov, et al. [62], such as to experimentally test restoration approaches and their effectiveness. For example, designed artificial structures called Reef Balls were deployed in the Netherlands Antilles [45], and three-dimensional printed ceramic tiles were deployed in Israel [44] to evaluate coral recruitment and thus explore the potential of built structures to serve as restoration tools. The abundance of evidence related to coral restoration is not surprising since our eligibility criteria required that ecological outcomes relate to coral or coral reef metrics, which are often measured in restoration projects. The moderate amount of evidence related to coastal protection mainly stemmed from laboratory experiments or modeling (e.g., [63–66]). with the exception of several field studies (e.g., [65, 67, 68]). This demonstrates a gap in scaling up built structures for coastal protection in coral ecosystems, similarly highlighted by Viehman, et al. [20]. Built structures related to environmental mitigation, such as in the aftermath of blast fishing, ship grounding, or dredging, accounted for fewer articles than expected. We hypothesize that this is because many environmental mitigation articles exist only in white papers. We were able to discover some of these through our literature solicitation to stakeholders (e.g., [69, 70]), but others likely exist that were not captured in our map. Few articles included interventions related to artwork or tourism and recreation, suggesting that when built structures are installed for these purposes, they may not be monitored at all, or may be monitored for other outcomes (social, economic, ecological – fish; for example) rather than ecological (coral) or physical outcomes (e.g., [71, 72]). Unintentionally deployed structures were also included in the map and provided a moderate amount of evidence; these often included ships sunk accidentally (e.g., [60, 73]); see Lemasson, et al. [74] for a meta-analysis on ecological effects of anthropogenic structures, including accidental shipwrecks.

#### Type and material of built structures

The distribution of evidence varied by built structure type, but the overwhelming majority of evidence was from artificial structures. This may relate to the historic use of artificial reefs for habitat enhancement in coral ecosystems and other ecosystems [25, 75, 76] and the rising amount of artificial or gray infrastructure across coastal habitats [77]. However, growing calls for incorporation of nature-inspired and gray-green (i.e., hybrid with both artificial and natural elements) designs have produced a moderate amount of evidence on hybrid structures to date [78–81]. For example, Miller, et al. [82] evaluated the ecological performance of hybrid restoration structures in the Florida Keys that were created from limestone and concrete, and Blakeway et al. [83] assessed the ecological performance of limestone and concrete reef modules following nearby land reclamation. Despite these case studies, the overall abundance of evidence on hybrid structures was eight times less than that of artificial evidence. Natural structures had the least amount of evidence; when evidence existed, it usually related to the introduction of structures of geological origin, such as limestone rocks or boulders (e.g., [84, 85]).

The most common built structure materials for artificial and hybrid interventions were concrete and metal, which matches a recent analysis of materials used in U.S. ocean artificial reefs managed by states [86]. We did find evidence that three-dimensional printed materials, often composed of plastic, are becoming more common [87–89]. This likely reflects recent innovations in the interdisciplinary engineering of built structures for coral restoration and related applications. There were multiple articles that evaluated proprietary structures, such as Reef Balls or Biorock, but overall, there was a diversity of structures that varied in size, shape, and material reflecting a mix of established structures and more recently developed structures.

#### Ecological and physical performance of built structures

Ecological evaluations of coral primarily focused on several outcomes with the complete absence of others. Outcomes such as coral mortality, coral growth, coral cover, and coral recruitment were studied repeatedly using metrics like percent cover, percent mortality, growth rate, and coral size and methods like video, photo, or visual transect surveys. The coral outcomes that were not evaluated were largely broader seascape outcomes, such as connectivity, or fine scale outcomes, such as microbiome, physiology, and calcification. This suggests that the evidence base on the ecological performance of built structures for coral is concentrated on population or community level outcomes and that gaps exist in seascape level, and in some cases, individual level outcomes. Multiple studies have been conducted on how built structures relate to fish population or community seascape patterns [90–92], and so this could be extended to examine coral seascape outcomes associated with built structures. Additionally, whereas the microbiome of epifauna on artificial reefs [93] and the microbiome of bacteria in the sediment surrounding artificial reefs [94] have been investigated, there is a lack of information on coral microbiomes on built structures. Our systematic map focused on ecological outcomes of built structures associated with coral, but there are other coral-reef related organisms and associated metrics across ecological scales that were beyond the scope of this map.

The pronounced evidence gaps in physical outcomes associated with built structures fits with recent calls for increased consideration of the physical performance of coral reefs, especially within coastal resilience frameworks [20]. Our systematic map findings reinforce the need for additional research on physical performance of built structures for reef-related applications. During article screening, we did find numerous studies that evaluated the physical performance of built structures in lab settings; however, because these studies often did not include corals, they did not meet the coral ecosystem requirement and were thus excluded (e.g., [95, 96]). This highlights a gap, where built structure physical performance is often monitored in lab settings without coral, whereas field studies typically focus on ecological coral-associated outcomes rather than physical outcomes. Future research could consider expanding our map to examine physical outcomes of built structures without coral in lab settings. Moreover, built structures are often evaluated solely for ecological or solely for physical outcomes; additional research could harness interdisciplinary collaborations to examine both ecological and physical outcomes simultaneously [20].

### Limitations of the map

We recognize several potential sources of bias in our systematic map. First, we restricted our search to English language due to resource constraints. Although our map includes evidence from 50 countries, we were unable to conduct full text screening on 76 articles because they were not in English. To help reduce bias, we ensured that our solicitation for contributed literature was shared outside of English-speaking countries with stakeholders from Spain, France, Monaco, Israel, Saudi Arabia, and several international organizations. Despite these measures, there is likely bias in our map towards English-speaking countries. Future efforts could broaden the evidence base by incorporating non-English language articles.

Second, we conducted single screening, which may have introduced bias into the systematic map. Single screening was necessary because of resource constraints and the high number of expected articles (∼ 20,000 after deduplication). We took several steps to maximize inter-reviewer consistency, including holding rigorous screening training sessions, evaluating inter-reviewer consistency with a random selection of articles, conducting double screening on a subset of articles, and flagging articles for a second opinion when a screener was unsure whether to include or exclude. These measures likely helped reduce bias.

Third, we used software with a machine learning algorithm to assist with title and abstract screening. The algorithm in the software Swift Active Screener incorporates screener feedback on which articles are marked as relevant or irrelevant [53]. The algorithm then ranks and shuffles unscreened articles in order of relevance for priority screening. We conducted screening until the software “recall rate” reached 95%. This approach has been tested and accepted in medical science evidence syntheses [97, 98]. It is possible, however, that using Swift Active Screener introduced bias into the systematic map if articles were overlooked by the algorithm and ranking system.

## Conclusion

### Implications for policy and management

Our map highlights several evidence clusters that can be used to help guide management decisions related to the use of built structures in coral reef ecosystems. For example, clusters of evidence on coral outcomes associated with designed artificial structures can be used by stakeholders including coral restoration managers and practitioners, environmental mitigation teams, coastal protection and resilience specialists, and artificial reef managers to help inform decisions. We caution, however, that the coral-related ecological performance of built structures is likely location-specific, as has been found for fish communities [75]. Managers should take utmost caution when extrapolating results from one geographic location to another or from one built structure material or type to another. Our map also reveals that the lack of information on the physical performance of coral reefs may impede the ability of the management community to make informed decisions. The type of decisions that may be impeded by the lack of evidence relate to the potential of built structures in coral ecosystems to provide coastal protection services, such as attenuating wave energy, reducing current magnitude, or decreasing storm surge. There is a great push, however, to utilize coral reef restoration for coastal protection [99, 100], which has large financial implications as it would allow for millions of dollars in pre-disaster mitigation or billions of dollars of post-disaster recovery funding for coral reef restoration. Despite this push, additional evidence stemming from lab and field studies will be needed to help better inform decisions on how to actively use built structures for coastal protection purposes. A systematic review and accompanying meta-analysis could also help determine the potential study bias and effect sizes associated with built structures and ecological and physical performance outcomes.

### Implications for research

Our systematic map findings can be used for a systematic review. There may be sufficient evidence to evaluate the ecological performance of artificial designed structures for coral mortality (*n* = 66), coral growth (*n* = 64), coral recruitment (*n* = 57), coral cover (*n* = 40), coral diversity (*n* = 26). A systematic review could also evaluate the ecological performance of coral diversity, recruitment, and cover across different types of built structures. There is likely not enough evidence to conduct a quantitative synthesis, such as a meta-analysis, on physical performance of built structures, as the highest concentration of evidence was 14 studies for outcomes related to waves. Future research could combine ecological and physical performance assessments of built structures and should focus on understanding effects of built structures across broader spatial and temporal scales. Key gaps remain in our understanding of how built structures perform across seascape scales, for example, but gaps also remain in our understanding of individual coral outcomes like microbiome and physiology. Future efforts can help strategically fill gaps in understanding physical outcomes of built structures by installing built structures as part of scaled field experiments and conducting in situ monitoring to determine changes in waves, currents, wind, and water level that may relate to the built structure. Study designs could compare different types of built structures but could also compare the presence vs. absence of built structures to help disentangle performance. Such studies could include teams with diverse subject matter expertise and skillsets to study both ecological and physical performance of built structures.

## Electronic supplementary material

Below is the link to the electronic supplementary material.

Supplementary Material 1

Supplementary Material 2

Supplementary Material 3

Supplementary Material 4

Supplementary Material 5

Supplementary Material 6

Supplementary Material 7

Supplementary Material 8

Supplementary Material 9

## Data Availability

Not applicable.
